# The effect of incentive spirometry in perioperative patients with lung cancer—a systematic review and meta-analysis

**DOI:** 10.1186/s12890-024-02878-1

**Published:** 2024-02-15

**Authors:** Yan Liang, Shaolin Chen, Jiamei Song, Ting Deng, Jinfen Yang, Yangyang Long, Lorna Kwai Ping Suen, Xu Luo

**Affiliations:** 1 Nursing Department, Affiliated Hospital of Zunyi Medical University, No. 149, Dalian Road, Huichuan District, Zunyi City, Guizhou Province 563000 China; 2 School of Nursing, Zunyi Medical University, No 6, Xuefu West Road, Zunyi City, Guizhou Province 563000 China; 3 School of Nursing, Tung Wah College, Kowloon, Hong Kong, SAR 999077 China; 4 School of Medical Informatics and Engineering, Zunyi Medical University, No 6, Xuefu West Road, Zunyi City, Guizhou Province 563000 China

**Keywords:** Incentive spirometry, Pulmonary rehabilitation, Lung cancer, Perioperative, Meta-analysis

## Abstract

**Background:**

Incentive spirometry (IS) as a routine respiratory therapy during the perioperative period has been widely used in clinical practice. However, the impact of IS on patients with perioperative lung cancer remains controversial. This review aimed to evaluate the efficacy of IS in perioperative pulmonary rehabilitation for patients with lung cancer.

**Methods:**

Cochrane Library, PubMed, Web of Science, Ovid, CINAHL, Chinese National Knowledge Infrastructure, Weipu, and Wanfang Databases were searched from inception to 30 November 2023. Only randomized controlled trials were included in this systematic review. The PRISMA checklist served as the guidance for conducting this review. The quality assessment of the included studies was assessed by the Cochrane risk-of-bias tool. The meta-analysis was carried out utilizing Review Manager 5.4. Furthermore, sensitivity analysis and subgroup analysis were also performed.

**Results:**

Nine studies recruited 1209 patients met our inclusion criteria. IS combined with other respiratory therapy techniques was observed to reduce the incidence of postoperative pulmonary complications, enhance pulmonary function, curtail the length of hospital stay, and lower the Borg score. Nevertheless, no improvements were found in the six-minute walk distance or quality of life score.

**Conclusions:**

Although IS demonstrates benefits as a component of comprehensive intervention measures for perioperative patients with lung cancer, it proves challenging to determine the precise impact of IS as a standalone component within the comprehensive intervention measures. Therefore, further researches are required to better understand the effectiveness of IS isolation and its interactions when integrated with additional respiratory therapies for these patients.

**Clinical trial registration:**

PROSPERO, https://www.crd.york.ac.uk/prospero/, registry number: CRD42022321044.

**Supplementary Information:**

The online version contains supplementary material available at 10.1186/s12890-024-02878-1.

## Introduction

According to the Global Cancer Statistics 2020, lung cancer is the second most common cancer and the leading cause of cancer-related deaths. It is estimated that there are 2.2 million new cases and 1.8 million deaths, accounting for 11.4 and 18.0% of diagnosed cancers and deaths, respectively [1]. Surgery is still considered as the primary therapy for the majority of patients diagnosed with stage I-III non-small cell lung cancer (NSCLC) [2]. Nonetheless, about 40% of patients have experienced postoperative pulmonary complications (PPCs) because of surgical trauma and pulmonary pathophysiological alterations in the perioperative phase [3]. PPCs have not only led to fatalities in approximately 85% of these patients, but also played a significant role in prolonging hospital stays and readmissions to the intensive care unit (ICU) [4]. These complications are widely defined as pneumonia, atelectasis, pleural effusion, pneumothorax, respiratory tract infection, bronchospasm, respiratory failure requiring invasive or non-invasive mechanical ventilation and so on [5–7]. Therefore, it is vital for clinical practitioners implementing effective interventions to prevent the occurrence of PPCs.

The therapy of pulmonary expansion can enable patients to maintain an effective cough mechanism, promote the clearance of postoperative respiratory secretions. Incentive spirometry (IS) is a mechanical device that promotes lung expansion [8]. Its aim is to simulate natural sighs or yawns by encouraging patients to take long, slow, deep breaths, reducing pleural pressure, promoting pulmonary expansion, and promoting gas exchange [9]. While physiological evidence suggests that IS could potentially benefit lung re-expansion following surgery, there exists a certain level of controversy among studies regarding its impact on the incidence of PPCs and the length of hospital stays [10–12].

Although previous meta-analyses [13] have addressed the effect of IS in patients undergoing cardiac, thoracic, and upper abdominal surgeries, this study included a total of 31 articles, of which only 6 were relevant to thoracic surgery. Upon careful examination of these 6 studies, it becomes apparent that only 2 studies centered on lung resection [11, 14], 2 studies [12, 15] observed both lung and esophagus surgeries, while another 2 studies [16, 17] investigated the application of IS in the realm of abdominal surgery. However, owing to lung resection, the effect of IS may be distinguished from other thoracic or abdominal surgeries. Furthermore, certain valuable Chinese studies were not included in the meta-analysis [18–22]. Therefore, it is hard to draw a conclusion of the effect of IS on perioperative lung cancer surgery patients. The present study aimed to synthesize existing evidence to identify the impact of IS on the perioperative period of lung cancer surgery, to provide substantive evidence for clinical practitioners to implement IS into clinical practice, to improve the prognosis of these patients.

## Methods

This meta-analysis was performed in accordance with the PRISMA (Preferred Reporting Items for Systematic Reviews and Meta-Analyses) guidelines [23], and registered in PROSPERO(CRD42022321044).

### Eligibility and exclusion criteria

The inclusion criteria were according to PICOS (Participants, Intervention, Comparison, Outcomes and Study type): (1) Participants (P): adults (aged ≥18 years) who were diagnosed with lung cancer during the perioperative phase, (2) Intervention (I): the experimental group accepted IS alone or in combination with other physical therapies. (3) Comparison (C): the control group received routine care or other physical therapies. (4) Outcomes (O): PPCs, pulmonary function, the length of hospital stays (LOS), Borg score, the six-minute walk distance (6MWD) or quality of life (QoL). (5) Studies (S): randomized controlled trials. (6) Language: publications in either the Chinese or English language. Review articles, letters, comments, case reports, conference abstracts and full text unavailable were excluded. We also retrieved the references of included studies which were meticulously scrutinized to uncover other potentially eligible studies.

### Search strategy

We performed a computer-based search in the Cochrane Central Register of Randomized Controlled Trials, PubMed, Web of Science, Ovid, CINAHL, Chinese National Knowledge Infrastructure, Weipu and Wanfang Databases. The database entries were searched from inception to 30 November 2023. The details of the search strategy were provided in Supplementary Material 1. 

### Study selection

Two authors (YL, JMS) individually screened the available studies. Verification of eligibility was determined based on information from the title and abstract, then we assessed the full text of potential studies to identify if they fitted the inclusion criteria. Decisions by the 2 authors were compared and any discrepancies were resolved by a third author (SLC).

### Data extraction

Data extraction was performed by 2 authors (YL, JMS). The following data were extracted: authors, publication year, journal, the characteristics of population, sample size, primary and secondary outcomes, duration and frequency of intervention, and so on.

### Quality appraisal

The risk of bias and quality of the included studies were assessed using the Cochrane risk of bias assessment tool [24]. The tool addresses 7 specific domains of potential bias: sequence generation, allocation concealment, blinding of participants and personnel, blinding of outcome assessment, incomplete outcome data, selective outcome reporting, and other biases. Risk of bias assessment was performed for all the included studies individually by 2 authors (YL, JMS); A third author (SLC) was available to resolve any disagreements.

### Statistical analysis

Review Manager 5.4 was employed for statistical analysis and to generate forest plots. The pooled estimates of intervention effect for dichotomous outcomes were quantified using the odds ratio (OR) with a 95% confidence interval (95% CI), while the mean difference (MD) with a 95% CI was utilized to quantify continuous outcomes. Forest plots were created to elucidate the effect size. We conducted a comparison between the intervention and control groups, and employed the following indicators of the intervention’s effect: The odds ratio (OR) with a 95% confidence interval (95% CI) was utilized to quantify the effect of intervention on PPCs, while the mean difference (MD) with a 95% CI was utilized to summarize the average values with standard deviations for pulmonary function, Borg score, length of stay (LOS), and quality of life (QoL) score for other outcome measures.

The statistical heterogeneity of intervention effects was evaluated using the *I*^*2*^ test and Cochran’s Q test. In instances where heterogeneity was significant (*I*^*2*^ > 50%), a random effects model was employed; otherwise, a fixed-effect model was utilized. We performed a sensitivity analysis to assess the stability of the outcome and to identify the source of heterogeneity. Some studies incorporated IS as a component of the intervention To evaluate the effectiveness of the intervention, which was predominantly centred on IS, in reducing postoperative pulmonary complications, we conducted a sub-group analysis on the implementation of IS combined with other respiratory therapy techniques. In order to explore the impact of IS and various interventions on the key pulmonary outcomes across different countries, we undertaken a sub-group analysis incorporating studies conducted in China and other countries. Publication bias was evaluated using funnel plots.

## Results

### Study selection

**Fig. 1:**
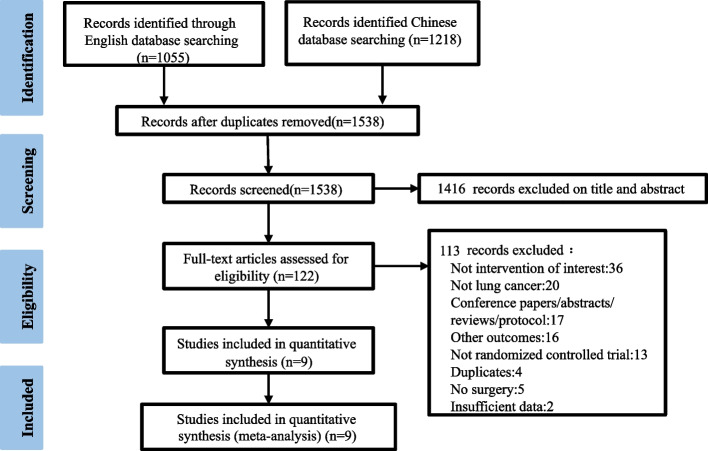
PRISMA flow chart of study selection

A total of 2273 studies were initially retrieved, comprising 1055 English records and 1218 Chinese records respectively. After the removal of duplicates, 1538 records were remained. 1416 articles were excluded after screening titles and abstracts, and 122 articles were retained for the full-text analysis. Finally, nine studies involving 1209 lung cancer patients were included in the meta- analysis [11, 14, 18–22, 25, 26] (Fig. 1 PRISMA flow chart of study selection).

### Study characteristics

All encompassed studies were RCTs with single-center design, six trials were conducted in China [18–22, 26], and the remaining trials were performed in UK [11], South Korea [25] and Canada [14]. Among the eligible RCTs, one study entailed preoperative intervention [21], while five studies involved postoperative intervention [11, 14, 18, 22, 25]. Additionally, three trials encompassed intervention during the perioperative period [19, 20, 26]. The duration of intervention ranged from 1 to 4 weeks. However, various studies utilized different interventions in control or intervention groups. Two studies [18, 25] compared IS with or without the combination of other devices, while two studies^[14, 19]^assessed the effects of routine physiotherapy with or without IS. Additionally, three studies [20, 22, 26] compared IS combined with other devices to breathing training, one study [11] compared IS with thoracic expansion exercises, and another [21] compared IS with routine breathing training. The details of all included studies were summarized in Table 1 (Table 1 The characteristics of the included studies).

**Table 1:** The Characteristics Of The Included Studies

Study	Country	Surgical Procedures	Pre/post/peri	Sex, M/F, C/T	Age(years), C/T	Patients, C/T	Intervention	Control	Frequency	Length of Intervention	Outcomes
Paula Agostini et al., 2013 [11]	UK	Thoracotomy	post	41/47 45/47	70 ± 9 65 ± 14	180 (88/92)	IS	Thoracic expansion exercises	Postoperative day1: Twice daily (10 repetitions) Postoperative Day2 continue until discharge: 10 times hourly while awake	Postoperative day1 to discharge	(1), (2) and (3)
Y. J. Cho et al., 2014 [25]	South Korea	VATS	post	21/18 22/17	56.5 ± 9.8 56.4 ± 10.5	78 (39/39)	IS+acapella	IS	10 deep breaths with the IS device at least every 2 h while awake	Postoperative day1	(2) and (3)
Peter R. A. et al., 2018 [14]	Canada	Thoracotomy+VATS	post	102/90 91/104	67.5 ± 10.4 66.6 ± 12.1	387 (192/195)	IS + physiotherapy	- routine physiotherapy	10 deep breaths with the IS device every waking hour	30 days after surgery	(1) and (3)
ZHENG Lina et al., 2019 [19]	China	NR	peri	38/13 36/10	64.84 ± 8.61 62.91 ± 9.33	97 (51/46)	IS + physiotherapy	routine physiotherapy	4 times a day, 20 minutes each time	7 days before surgery and 7 days after surgery	(1), (2), (4) and (5)
LIU Xiang et al., 2022 [21]	China	NR	Pre	24 /25 26 /23	58.72 ± 10.34 57.16 ± 9.68	98 (49/49)	IS	routine breathing training	4 times a day, 10–15 minutes each time	7 days before surgery	(1), (2), (4) and (5)
WANG Jicui et al., 2022 [20]	China	VATS	peri	13/17 15/15	51.29 ± 9.56 55.42 ± 7.45	60 (30/30)	IS+vibration expectoration vest	deep breathing training + cough and expectoration training	4 times a day	7 days before surgery and 7 days after surgery	(1), (2)
ZHANG Jie et al., 2021 [26]	China	VATS	peri	32/26 30/28	54.31 ± 8.07 56.23 ± 6.78	116 (58/58)	IS+vibration expectoration vest	deep breathing training + cough and expectoration training	4 times a day	3 days before surgery and 7 days after surgery	(1), (2)
ZHANG Xinna et al., 2021 [22]	China	VATS	post	27/16 29/15	66.35 ± 6.30 66.27 ± 5.63	87 (43/44)	IS+OPEPD	ACBT	4 times a day, 10–15 minutes each time	5 days after surgery	(1), (4), (5) and (6)
ZHU Li et al., 2022 [18]	China	VATS	post	33/20 34/19	59.74 ± 5.62 59.25 ± 8.93	106 (53/53)	IS+vibration expectoration vest	IS	4 times a day, 15–20 minutes each time	14 days after surgery	(1), (2) and (6)

*ACBT* Active cycle of breathing techniques, *C/T* Control group/treatment group, *IS* Incentive spirometry, *M/F* Male/female, *NR* Not reported, *OPEPD* Oscillatory positive expiratory pressure device, *Pre/Post/Peri* Preoperative/ Postoperative/Perioperative, *RCT* Randomized controlled trial, *VATS* Video-assisted thoracoscopic surgery: (1) postoperative pulmonary complications (PPCs); (2) pulmonary function; (3) length of stay (LOS); (4) Borg score; (5) 6-min walk distance(6-MWD); (6) quality of life (QoL)

### Quality assessment

**Fig. 2:**
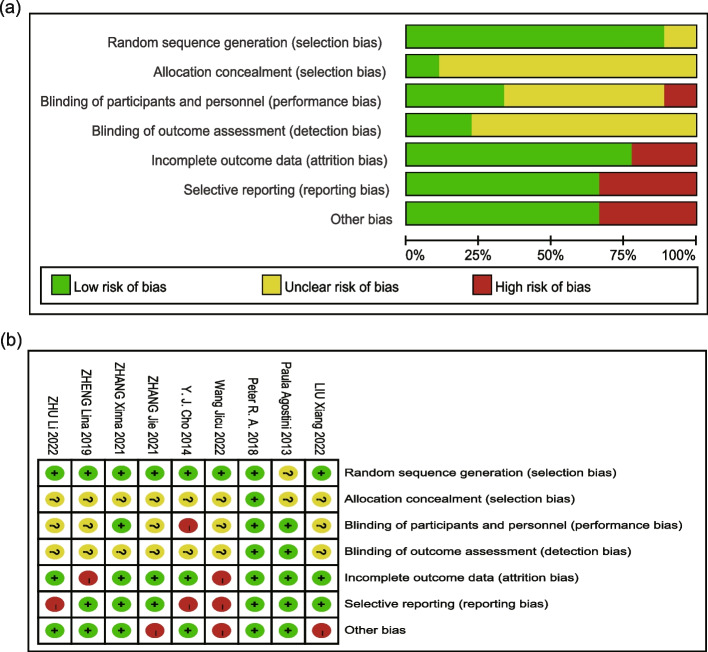
Risk of Bias

All trials endeavored to randomize patients into intervention group and control group, but some of them failed to specify the exact details of the randomization procedures. Due to the nature of IS, it was difficult to achieve blinding of patients and personnel, causing studies with low risk of bias to be rare. The results of the risk of bias evaluation for the included trials were summarized in the risk of bias graph (Fig. 2 Risk of bias).

### Outcomes of the meta-analysis

Eight studies reported PPCs [14, 18–22, 25, 26](the primary outcome measures in this analysis), seven studies focused on pulmonary function [11, 19–22, 25, 26], three studies noted Borg score and 6-MWD [19, 21, 22], and three studies documented the LOS [11, 14, 25], two studies recorded the QoL [18, 22].

#### PPCs

**Fig. 3:**
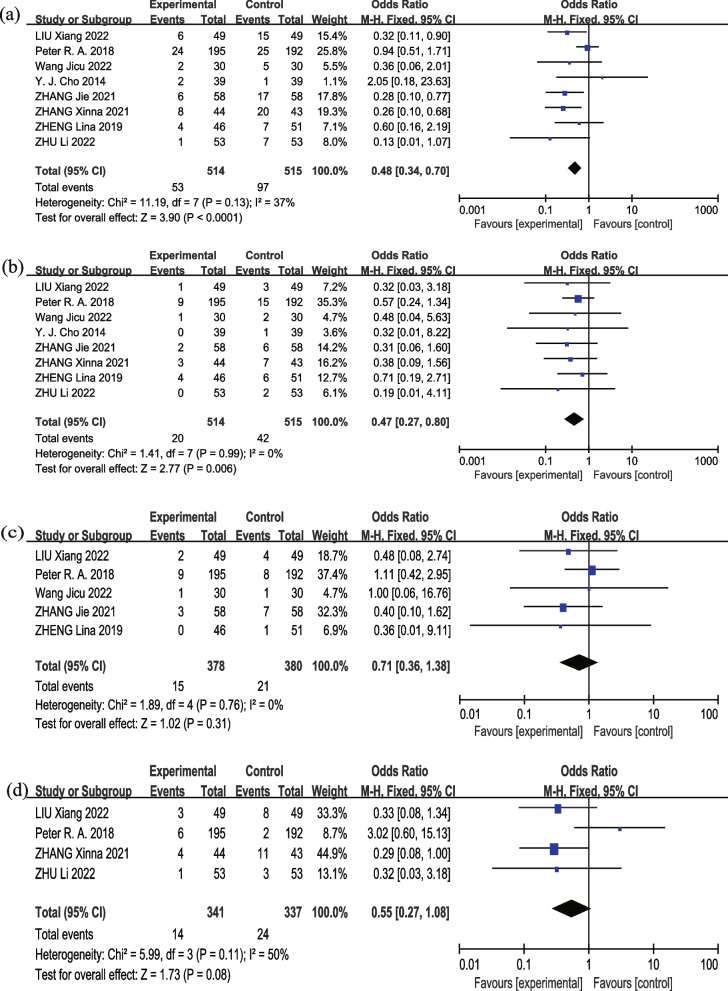
Forest plot of PPCs. **a **postoperative pulmonary complications (PPCs); **b **pneumonia; **c **atelectasis; **d **respiratory insufficiency

Eight studies [14, 18–22, 25, 26] investigated the effect of intervention on the rate of PPCs. There was moderate heterogeneity (*I*^*2*^ = 37%, *p* = 0.13) among studies, a fixed-effect model was implemented, and it demonstrated that intervention group could decrease the PPCs compared with control group (OR = 0.48, 95% *CI*: 0.34–0.70, *p* < 0.0001) (Fig. 3(a) Forest plot of PPCs). Eight studies [14, 18–22, 25, 26] explored the risk of pneumonia, indicating that IS combined with other respiratory therapy techniques could decrease the rate of pneumonia (*OR* = 0.47, 95% *CI*: 0.27–0.80, *p* = 0.006) (Fig. 3(b) Forest plot of pneumonia). Five studies [14, 19–21, 26] reported the risk of atelectasis. There was no heterogeneity *(I*^*2*^ = 0, *p* = 0.76) among these studies, and showed no statistically significant difference (*OR* = 0.71, 95% *CI*: 0.36–1.38, *p* = 0.31) (Fig. 3(c) Forest plot of atelectasis). Four studies [14, 18, 21, 22] analyzed the risk of respiratory insufficiency. There was a significant heterogeneity (*I*^*2*^ = 50%, *p* = 0.11) between the intervention and control groups (*OR* = 0.55, 95% *CI*: 0.27–1.08, *p* = 0.08) (Fig. 3(d) Forest plot of respiratory insufficiency), demonstrating a lack of statistically significant difference between the two groups.

To evaluate the dependability and robustness of the meta-analysis, a sensitivity analysis was performed. For PPCs, the heterogeneity significantly decreased (*I*^*2*^ = 0%, *p* = 0.66) when we removed Peter R. A. et al.’s study [14]. The adjusted pooled estimates, however, had not changed significantly (*OR* = 0.33, 95% *CI*: 0.20–0.53, *p* < 0.000001), manifesting that this investigation served as the primary cause of the heterogeneity. The examination implied that the outcomes in this meta-analysis were fairly robust. The heterogeneity of respiratory insufficiency significantly decreased (*I*^*2*^ = 0, *p* = 0.99) when we removed Peter R. A. et al.’s study [14], but the adjusted pooled estimates changed significantly (*OR* = 0.31, 95% *CI*: 0.13–0.73, *p* = 0.008). The elucidation indicated that the stability of this meta-analyses was inadequate to demonstrate the statistical significance of the difference in risk of postoperative respiratory dysfunction between the two groups.

#### Pulmonary function

**Fig. 4:**
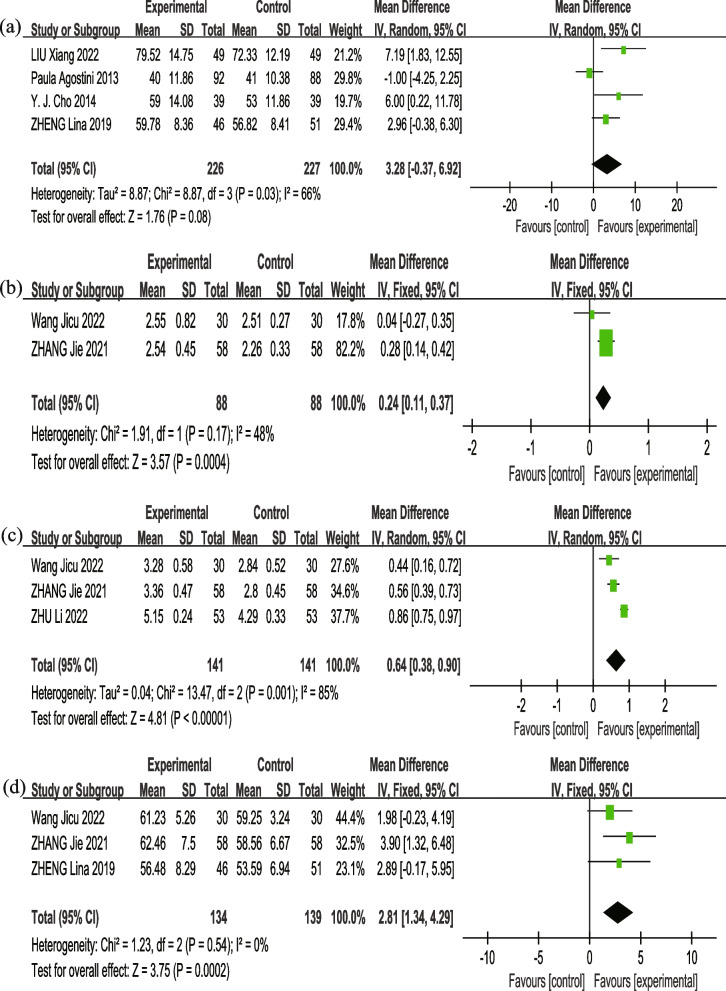
Forest plot of pulmonary function. **a** FEV1% within 7 days after surgery; **b** FEV1(L) within 7 days after surgery; **c** FVC% within 7–14 days after surgery; **d** MVV within 7 days after surgery

Seven studies [11, 19–22, 25, 26] assessed pulmonary function within 7 days post-surgery in patients. Four studies [11, 19, 21, 25] on 453 patients tested forced expiratory volume in the first second percentage of predicted normal values (FEV1%), and IS combined with other respiratory therapy techniques did not demonstrate significant enhancements in FEV1% predicted (*MD* = 3.28, 95% *CI*: − 0.37–6.92, *p* = 0.08, *I*^*2*^ = 66%) (Fig. 4(a) Forest plot of FEV1% within 7 days after surgery). With the exception of Agostini et al.’s study [11] that did not have a positive impact on FEV1%, the remaining three studies [19, 21, 25] all increased FEV1% to varying degrees. The exclusion of Agostini et al.’s study [11] significantly reduced the heterogeneity of anticipated FEV1% values (*I*^*2*^ = 2%, *p* = 0.36), resulting in substantial changes in the adapted consolidated approximations (*MD* = 4.53, 95% *CI*: 1.94–7.11, *p* = 0.0006), which further suggests that this study was the main driver of the heterogeneity. Two studies [20, 26] delineated the actual values of FEV1, validating the efficacy of IS combined with other respiratory therapy techniques in enhancing FEV1 values (*MD* = 0.24, 95% *CI*: 0.11–0.37, *p* = 0.0004) (Fig. 4(b) Forest plot of FEV1(L) within 7 days after surgery). Three studies [18, 20, 26] reported predicted forced vital capacity (predicted FVC%), and showed that the experimental group obviously benefitted in FVC% with significant heterogeneity (*MD* = 0.64, 95% *CI*: 0.38–0.90, *P* < 0.00001, *I*^*2*^ = 85%) (Fig. 4(c) Forest plot of FVC% within 7–14 days after surgery). Our analysis suggested that the observed differences may be attributed to variations in time of measurement. Among these three studies, ZHU Li et al.’s research [18] was conducted 2 weeks after surgery, while the other two [20, 26] were conducted within 1 week after surgery. Thus, ZHU Li et al.’s study [18] reported a higher FVC% compared to the other two studies, mainly contributing to the heterogeneity observed. Three studies [19, 20, 26] explored maximal voluntary ventilation (MVV), and suggested that the experimental group had obvious benefits in MVV with no heterogeneity (*MD* = 2.81, 95% *CI*: 1.34–4.29, *P* = 0.0002, *I*^*2*^ = 0%) (Fig. 4(d) Forest plot of MVV within 7 days after surgery).

#### Other outcomes

**Fig. 5:**
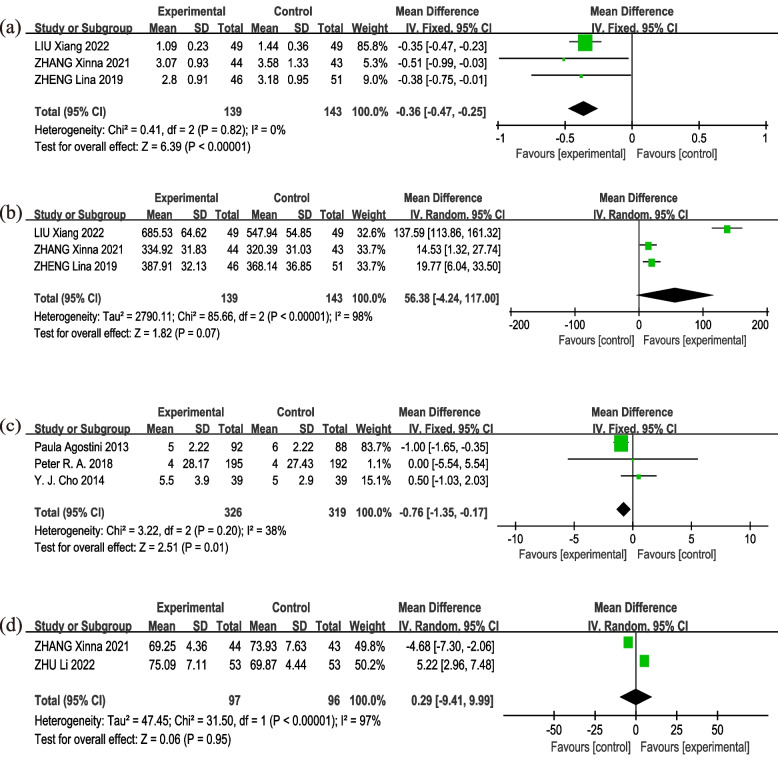
Forest plot of other outcomes. **a **Borg scores; **b **six-minute walk distance(6-MWD); **c **the length of hospital stays (LOS); **d **quality of life (QoL)

Three studies [19, 21, 22] utilized the Borg scale to evaluate the level of respiratory distress in the two patient groups within one-week post-surgery. There was no heterogeneity (*I*^*2*^ = 0%, *p* = 0.82) in Borg scale (*MD* = − 0.36, 95% *CI*: − 0.47 – − 0.25, *p* < 0.00001) (Fig. 5(a) Forest plot of Borg scores), thereby substantiating the effectiveness of intervention group in diminishing Borg scores. Three studies [19, 21, 22] reported the effect of intervention on the 6-MWD. There was significant heterogeneity (*I*^*2*^ = 98%, *p* < 0.00001), as a result, we used a random effects model for analysis, which revealed no significant difference between the two groups (*MD* = 56.38, 95% *CI*: − 4.24–117.00, *p* = 0.07) (Fig. 5(b) Forest plot of 6-MWD). There was a significant decrease in heterogeneity (*I*^*2*^ = 0%, *p* = 0.59) following the removal of LIU Xiang et al.’s study [21], resulting in substantial changes in the adapted consolidated approximations (*MD* = 17.05, 95% *CI*: 7.53–26.57, *p* = 0.0004), which further suggested that this study was the primary contributor to the heterogeneity.

Three studies [11, 14, 25] delineated the impact of intervention on the LOS. There was moderate heterogeneity in LOS (*I*^*2*^ = 38%, *p* = 0.20), implying that the intervention is proficient in curtailing the LOS (*MD* = -0.76, 95% *CI*: − 1.35–-0.17, *p* = 0.01) (Fig. 5(c) Forest plot of LOS). The analysis of two studies [18, 22] demonstrated evidence of high heterogeneity on the QoL (*I*^*2*^ = 97%, *p* < 00001). Therefore, we selected the random effects model for the analysis (*MD* = 0.29. 95% *CI*: − 9.41–9.99, *p* = 0.95) (Fig. 5(d) Forest plot of QoL). The result showed that the QoL was not significantly different between two groups.

It is noteworthy that the studies conducted by Y. J. Cho and ZHU Li et al. encompassed the inclusion of IS in both the control and intervention groups. In order to further examine whether they would exert an impact on the outcomes, we also conducted a sensitivity analysis on this aspect. The findings demonstrate that, when compared to the preceding meta-analysis, there were no alterations in the direction of any of the research outcomes. This signifies that the impact of these two studies on the results is not significant, thereby attesting to the stability of the meta-analysis results.

### Subgroup analysis

**Fig. 6:**
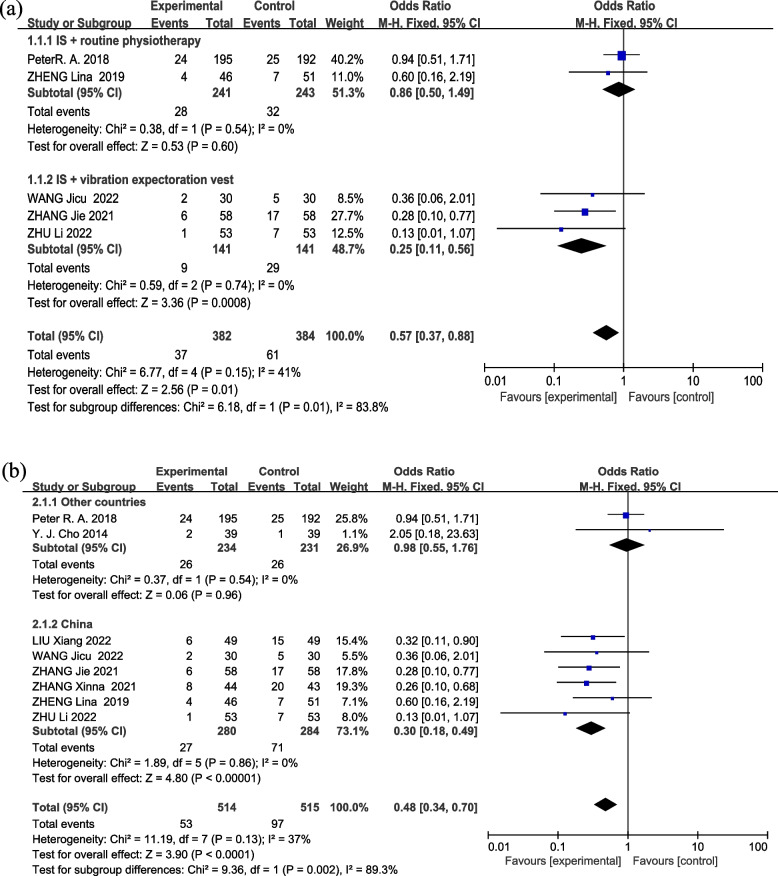
Forest plot of subgroup analysis of PPCs. (**a**) subgroup analysis of various interventions; (**b**) subgroup analysis of different countries

For subgroup analysis of PPCs of various interventions, we found that the subgroup of IS with routine physiotherapy had no difference between the two groups. Nevertheless, IS with vibration expectoration vest, a significant difference was noted between the two groups (Fig. 6(a) Forest plot of subgroup analysis of PPCs of various interventions).

For subgroup analysis of PPCs of different countries, it showed that the subgroup of other countries exhibited no difference between the two groups. However, in the subgroup of China, there was a significant difference between the two groups (Fig. 6(b) Forest plot of subgroup analysis of PPCs of different countries).

### Publication bias

**Fig. 7:**
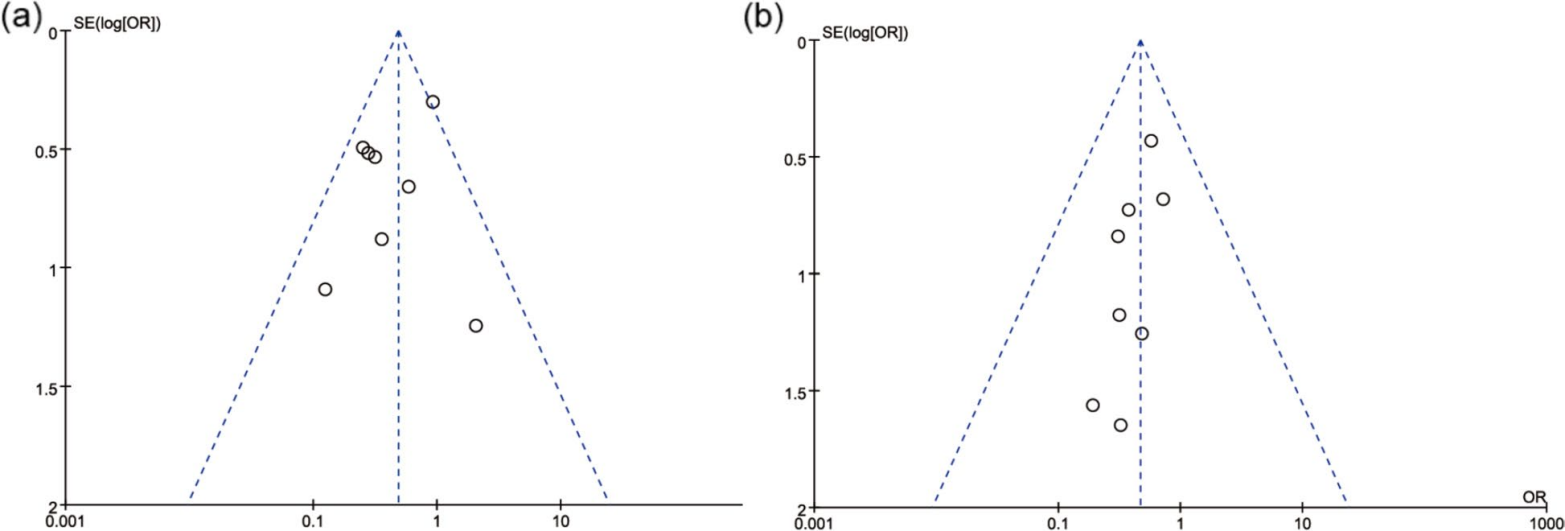
Funnel plot. (**a**) Funnel plot of PPCs; (**b**) Funnel plot of pneumonia

Herein, the incidence of PPCs and pneumonia was analyzed using funnel plots. The funnel plot of PPCs, however, was not entirely symmetrical, indicating the possibility of some degree of publication bias (Fig. 7(a, b) Funnel plot of PPCs; (b) Funnel plot of pneumonia).

## Discussion

To our knowledge, this is the first systematic review that solely comprises RCT data to analyze the effects of IS alone or combined with other respiratory therapy techniques on perioperative lung cancer patients. The findings of this study indicate that IS combined with other respiratory therapy techniques may provide several benefits to lung cancer patients undergoing surgery, as it can reduce PPCs and LOS, improve pulmonary function, and decrease the Borg score. However, due to the limited number of RCTs and the restricted set of outcome measures used in this analysis, it is challenging to determine the efficacy of IS alone on perioperative patients with lung cancer. The nine studies included had varying intervention timelines, with preoperative interventions lasting 1 week [21], postoperative interventions lasting from 5 days to 1 month [11, 14, 18, 22, 25], and perioperative interventions lasting 2 weeks [19, 20, 26]. The intervention modalities in our analysis differed as well. Therefore, we believe that further comprehensive evaluation is necessary to assess the impact of IS alone on perioperative lung cancer patients.

The incidence of PPCs leads to an escalated mortality rate, prolonged hospitalization, and augmented readmission rate [27, 28]. Therefore, it is crucial for the prognosis of patients to effectively prevent PPCs after lung cancer surgery. The utilization of IS in pulmonary rehabilitation serves as a valuable instrument in respiratory exercise, with the aim of mitigating or reducing PPCs and facilitating pulmonary rehabilitation [28]. Some studies suggest that IS may be more effective than non-interventional physical therapy [10, 29]. Our meta-analysis reveals that IS combined with other respiratory therapy techniques can decrease the incidence of overall PPCs (Fig. 3(a) Forest plot of PPCs). However, only one study each was available for IS alone, IS with acapella or OPEPD, and subgroup analyses are not feasible. Among these studies, IS with acapella did not reach statistical significance, IS with OPEPD showed a significant difference, and the odds ratio for IS alone had a 95% confidence interval approaching 0.9. Therefore, it is challenging to determine the impact of IS in isolation or in combination with acapella or OPEPD on PPCs. Additionally, in subgroup analysis, no significant difference was found in IS with routine physiotherapy, while a significant positive impact was observed with IS combined with a vibration expectoration vest (Fig. 6(a) Forest plot of subgroup analysis of PPCs of various interventions). The differences observed may stem from limited included studies and methodological variations, including differences in IS intervention implementation and sample size discrepancies across subgroups. Further explorations are needed to understand the potential independent effects of IS, and synergistic or antagonistic effects resulting from the integration of IS with other respiratory therapy techniques.

In addition, the subgroup analysis of PPCs in China and other countries showed difference (Fig. 6(b) Forest plot of subgroup analysis of PPCs of different countries). Firstly, this disparity may be attributed to a multitude of factors such as patient characteristics, environmental elements, genetic diversity, and so forth, existing within different cities. Secondly, potential disparate treatment and care measures across countries may influence postoperative outcomes, stemming from varied medical practices. Other factors, including sample size, the quality of study design, and characteristics of the study population, may also exert an impact on the results. It is noteworthy that further research is imperative to ascertain and validate the explanation behind such disparities.

IS facilitates the augmentation of patients’ postoperative volitional respiratory capacity, enhancing alveolar gas exchange function by increasing respiratory muscle activity, thereby improving pulmonary capacity and ameliorating lung function [30]. It has been substantiated to ameliorate postoperative pulmonary functions in several studies. For instance, Kundra et al. [31] noted a noteworthy improvement in pulmonary function following preoperative IS (*P* < 0.05), moreover, preoperative IS was found to be more efficacious in preserving pulmonary functions compared to postoperative IS. A randomized trial investigating postoperative outcomes in patients who underwent laparotomy showed that both volume-oriented and flow-oriented IS effectively ameliorated pulmonary functions [32]. However, this review only demonstrated that IS combined with other respiratory therapy techniques can enhance pulmonary function in patients undergoing lung cancer surgery. It remains challenging to establish the isolated effect of IS on pulmonary function.

Nonetheless, although this study exhibited an improvement in FEV1 values, FVC%, and MVV due to intervention, there exists inadequate evidence to substantiate a significant improvement in FEV1% due to the significant heterogeneity among four studies. Two studies [11, 25] conducted the interventions after surgery, and found no statistically significant difference in FEV1% between the intervention group and the control group. On the other hand, the other two trials [19, 21], analyzed the effects of perioperative and preoperative intervention and identified significant improvements in FEV1% among patients.

The six-minute walk test (6MWT) is a highly valuable tool for assessing the pulmonary functional training capacity of individuals afflicted with pulmonary ailments, given its proximity to everyday life, simplistic terrain, ease of acceptance and implementation by patients, and superior ability to reflect the patient’s daily life capacity. As a result of these advantages, the 6MWT is widely utilized in clinical settings [33]. However, the meta-analysis found that IS did not improve 6MWD [19, 21, 22]. Through sensitivity analysis, we found that LIU Xiang et al. [21] article was a source of heterogeneity, as in his study, 6MWD after 1 week of surgery was significantly higher than in the other two studies, which may lead to bias. The 6MWT is typically combined with the Borg scale to evaluate the pulmonary functional capacity of patients. The results of this study exhibited that IS combined with other respiratory therapy techniques can reduce Borg score.

The results of this study showed that IS combined with other respiratory therapy techniques can effectively reduce hospitalization time in lung cancer surgery patients. However, there is moderate heterogeneity among studies, possibly due to the fact that studies are implemented in different countries, and there are significant differences in routine hospitalization time for surgical patients in the intervention measures. Similar studies, such as Oliveira et al. ‘s research [34] demonstrated that respiratory muscle training improved pulmonary function and shortened postoperative hospital stays. Two studies [18, 22] reported the effects of intervention on postoperative QoL, both of which evaluated postoperative QoL in lung cancer patients using the revised version of the quality of life questionnaire for lung cancer patients. The analysis found that IS did not improve postoperative quality of life scores in lung cancer patients.

We discovered that despite the simplicity, accessibility, and cost-effectiveness of IS, most studies did not focus on the compliance and standardization of IS. As we know, compliance is crucial for the effectiveness of interventions. Inadequate training and insufficient self-administration of IS may lead to unresolved postoperative complications. A nationwide survey of healthcare providers found that out of 1681 respondents, 86% believed that patient compliance was poor. The primary reasons are patients forgetting how to utilize IS devices (83.5%; 1404 respondents), ineffectively using them (74.4%; 1251 respondents), and insufficient frequency of usage (70.7%; 1188 respondents) [35]. Therefore, it is imperative that we should enhance patient compliance with IS and provide standardized instruction in the future trails. The guidelines suggest that instructing clients and other healthcare providers in the technique of IS may facilitate the patient’s proper usage and promote adherence [36]. Furthermore, a potential strategy to enhance adherence and technique could involve educating patients using the device prior to surgery, instead of postoperative, to when the patient may be unable to effectively concentrate.

Although our meta-analysis shows strong evidence, however, there are several limitations. Firstly, we only included Chinese and English languages studies. Secondly, it indicated a certain degree of publication bias. Thirdly, there are many factors that may lead to clinical heterogeneity, including divergent characteristics of the participants, intervention measures, and study designs. Most clinical trials failed to blind patients and participants, as well as outcome assessment variables, which may also lead to methodological heterogeneity. Finally, various studies employed different interventions in control or intervention groups, highlighting a lack of standardized implementation of IS across these studies, and few eligible studies were included for each outcome indicator in same interventions. We have also attempted to analyze the effects of IS alone. However, this approach carries significant limitations. It proves challenging to ascertain the individual efficacy of IS. Therefore, further researches are needed to investigate this issue.

## Conclusion

In this meta-analysis, IS combined with other respiratory therapy techniques can reduce the incidence of PPCs, especially pneumonia, improve predicted FVC%, FEV1, and MVV values, as well as reduce postoperative Borg score and shorten hospitalization duration. However, the majority of research designs incorporate IS as an integral component of the intervention measures. Moreover, the specific impact of IS within the comprehensive intervention plan eludes extraction and quantification. Hence, it is challenging to ascertain the precise effects of the use of IS in isolation in this cohort. Future studies with large cohort should focus on exploring this issue, enhancing compliance, and facilitating optimal postoperative pulmonary rehabilitation in patients with perioperative lung cancer.

### Supplementary Information

**Additional file 1.**

## Data Availability

The dataset employed and/or scrutinized throughout the present investigation shall be obtainable upon a reasonable solicitation from the corresponding author, subsequent to the publication of the ultimate findings within a scholarly journal.

## Abbreviations

IS: Incentive spirometry
PPCs: Postoperative pulmonary complications
AARC: The American Association for Respiratory Care
NSCLC: Non small cell lung cancer
RCTs: Randomized controlled trials
SCLC: Small-cell lung cancer
LOS: The length of hospital stay
6MWD: The six-minute walk distance
QoL: Quality of life
OR: Odds ratio
CI: Confidence interval
MD: Mean difference
FEV1%: Forced expiratory volume in the first second percentage of predicted normal values
FVC: Forced vital capacity
MVV: Maximal voluntary ventilation
6MWT: The six-minute walk test
OPEPD: Oscillatory positive expiratory pressure device
