# Supplementary material for: The effect of incentive spirometry in perioperative patients with lung cancer—a systematic review and meta-analysis
Source: BMC Pulm Med. 2024 Feb 15;24:88. doi: 10.1186/s12890-024-02878-1 (PMC10870616; doi:10.1186/s12890-024-02878-1)
Supplement: Supplementary file 1 — Additional file 1. [file 12890_2024_2878_MOESM1_ESM.pdf]

Supplementary Material 1. Search string databases

Database	Language	Result	Date of search	Search string
Cochrane library	English	215	30/11/2023	TI,AB,KW: (Lung Neoplasms OR Lung Cancer OR Lung Tumor OR Lung Carcinoma OR Pulmonary Carcinoma OR Pulmonary Neoplasm OR Pulmonary Cancer OR NSCLC) AND (Incentive Spirometry OR Breathing Exercises OR Respiratory Training) AND (Perioperative OR Preoperative OR postoperative OR surgery OR pulmonary surgical procedures OR thoracic surgery OR pulmonary resection OR lung resection OR pneumonectomy)
CIINAHL	English	226	30/11/2023	TX(Lung Neoplasms OR Lung Cancer OR Lung Tumor OR Lung Carcinoma OR Pulmonary Carcinoma OR Pulmonary Neoplasm OR Pulmonary Cancer OR NSCLC) AND TX (Incentive Spirometry OR Breathing Exercises OR Respiratory Training) AND TX (Perioperative OR Preoperative OR postoperative OR surgery OR pulmonary surgical procedures OR thoracic surgery OR pulmonary resection OR lung resection OR pneumonectomy)
Ovid	English	343	30/11/2023	((Lung Neoplasms or Lung Cancer or Lung Tumor or Lung Carcinoma or Pulmonary Carcinoma or Pulmonary Neoplasm or Pulmonary Cancer or NSCLC) and (Incentive Spirometry or Breathing Exercises or Respiratory Training) and (Perioperative or Preoperative or postoperative or surgery or pulmonary surgical procedures or thoracic surgery or pulmonary resection or lung resection or pneumonectomy)).tw.
PubMed	English	70	30/11/2023	(("Lung Neoplasms"[Mesh] OR Lung Neoplasms[Title/Abstract] OR Lung Cancer[Title/Abstract] OR Lung Tumor[Title/Abstract] OR Lung Carcinoma[Title/Abstract] OR Pulmonary Carcinoma[Title/Abstract] OR Pulmonary Neoplasm[Title/Abstract] OR Pulmonary Cancer[Title/Abstract] OR NSCLC[Title/Abstract]) AND ((((Incentive Spirometry[Text Word] OR Breathing Exercises[Text Word] OR Respiratory Training[Text Word]))OR (breathing exercises[MeSH Terms])))) AND (Perioperative[Title/Abstract] OR Preoperative[Title/Abstract] OR postoperative[Title/Abstract] OR surgery[Title/Abstract] OR pulmonary surgical procedures[Title/Abstract] OR thoracic surgery[Title/Abstract] OR pulmonary resection[Title/Abstract] OR lung resection[Title/Abstract]

				OR pneumonectomy[Title/Abstract])
Web of Science	English	201	30/11/2023	TOPIC:(Lung Neoplasms OR Lung Cancer OR Lung Tumor OR Lung Carcinoma OR Pulmonary Carcinoma OR Pulmonary Neoplasm OR Pulmonary Cancer OR NSCLC) AND TOPIC: (Incentive Spirometry OR Breathing Exercises OR Respiratory Training) AND TOPIC: (Perioperative OR Preoperative OR postoperative OR surgery OR pulmonary surgical procedures OR thoracic surgery OR pulmonary resection OR lung resection OR pneumonectomy)
Chinese National Knowledge Infrastructure	Chinese	475	30/11/2023	(TI,AB,KW=Lung Neoplasms + Lung Cancer + Lung Tumor + Lung Carcinoma + Pulmonary Carcinoma + Pulmonary Neoplasm + Pulmonary Cancer + NSCLC) AND (TI,AB,KW= Incentive Spirometry + Breathing Exercises+ Respiratory Training) AND (TI,AB,KW=Perioperative + Preoperative + postoperative + surgery + pulmonary surgical procedures + thoracic surgery + pulmonary resection + lung resection + pneumonectomy)
Wanfang	Chinese	514	30/11/2023	TI,AB,KW: (Lung Neoplasms OR Lung Cancer OR Lung Tumor OR Lung Carcinoma OR Pulmonary Carcinoma OR Pulmonary Neoplasm OR Pulmonary Cancer OR NSCLC) AND (Incentive Spirometry OR Breathing Exercises OR Respiratory Training) AND (Perioperative OR Preoperative OR postoperative OR surgery OR pulmonary surgical procedures OR thoracic surgery OR pulmonary resection OR lung resection OR pneumonectomy)
Weipu	Chinese	229	30/11/2023	TI,KW: (Lung Neoplasms OR Lung Cancer OR Lung Tumor OR Lung Carcinoma OR Pulmonary Carcinoma OR Pulmonary Neoplasm OR Pulmonary Cancer OR NSCLC) AND (Incentive Spirometry OR Breathing Exercises OR Respiratory Training) AND (Perioperative OR Preoperative OR postoperative OR surgery OR pulmonary surgical procedures OR thoracic surgery OR pulmonary resection OR lung resection OR pneumonectomy)
